# Mechanical Hatching as a Therapeutic Intervention for Improving Implantation Rate in a 32-Year-Old Female With Recurrent Implantation Failures: A Case Report

**DOI:** 10.7759/cureus.53709

**Published:** 2024-02-06

**Authors:** Rabia Kakatikar, Pranita A Bawaskar, Ujwal Gajbe, Akash More, Nancy Nair

**Affiliations:** 1 Clinical Embryology, School of Allied Health Sciences, Datta Meghe Institute of Higher Education and Research (DMIHER), Wardha, IND; 2 Anatomy, Datta Meghe Medical College, Datta Meghe Institute of Higher Education and Research (DMIHER), Wardha, IND; 3 Anatomy, Jawaharlal Nehru Medical College, Datta Meghe Institute of Higher Education and Research (DMIHER), Wardha, IND

**Keywords:** recurrent implantation failures, implantation, endometrial receptivity analysis, mechanical hatching, consanguineous marriage, zona pellucida

## Abstract

This case report explores the application of mechanical hatching as a successful intervention in the treatment of primary infertility for a couple with a consanguineous marriage history and recurrent implantation failure. A 32-year-old female patient and her 37-year-old spouse, after six years of unsuccessful attempts to conceive, underwent multiple intrauterine insemination (IUI) and in vitro fertilization (IVF) and embryo transfer (ET) cycles without success. Normal parameters were observed in semen analysis and hormone tests for the male and female partners, respectively. Despite a series of failed assisted reproductive technology (ART) procedures, the implementation of mechanical hatching using partial zona dissection (PZD) pipettes led to a positive pregnancy outcome. The case underscores the potential efficacy of individualized approaches, specifically mechanical hatching, in addressing challenges associated with implantation failure, offering hope to couples facing infertility issues.

## Introduction

Clinical infertility is defined by the World Health Organization (WHO) and International Committee Monitoring Assisted Reproductive Technologies (ICMART) as the absence of pregnancy following 12 months or more of regular, unprotected sexual activity [1]. Primary infertility is the term used to describe couples who have had sexual contact for at least a year without using birth control yet are unable to conceive. Secondary infertility is a couple’s inability to conceive after at least one successful pregnancy [2]. Conversely, one or both spouses may be responsible for a couple’s reproductive issues. These couples benefit from assisted reproductive technology (ART) to get pregnant. Over the past 10 years, ARTs have been vital to treating infertility [3]. To increase the success rate of in vitro fertilization (IVF) and embryo transfer (ET) or intracytoplasmic sperm injection (ICSI), ovarian stimulation, fertilization, culture, and ET procedures have been improved [4].

The underlying cause of infertility, the ages of the partners, the health of the women’s reproduction system, and any potential genetic factors can impact the success rates of ARTs such as intrauterine insemination (IUI) and IVF [5,6]. Female fertility is influenced by various factors, both biological and environmental. Age is a significant factor, with fertility declining notably after the age of 35. Ovulation disorders such as polycystic ovary syndrome, hormonal imbalances, uterine and cervical abnormalities, fallopian tube damage, endometriosis, and primary ovarian insufficiency can impact fertility. Additionally, lifestyle factors such as nutrition, exercise, and stress, along with poor diet, excessive alcohol, smoking, and obesity, play crucial roles. Environmental factors such as exposure to chemicals may also affect fertility [4,7]. The close genetic bond between spouses may increase the risk of passing on specific genetic traits that might obstruct adequate conception and pregnancy in consanguineous couples who have reproductive problems [7]. A consanguineous marriage is a union of two people with a close blood relationship [8]. It usually refers to first cousins or even more immediate relatives. Regardless of whether a couple has a history of infertility, it is critical to understand that IVF and IUI success rates might differ for various couples.

Failing to create a sticky matrix among the blastocyst and endometrium is a failed implantation. Numerous variables can affect implantation, a very complex biological process that includes embryo hatching, localization, attachment, and invasion. A crucial stage for a successful implantation is hatching [9]. It has been demonstrated that cryopreservation and in vitro cultivation can thicken or harden the zona pellucida (ZP), impairing its capacity to hatch. As a solution, the ZP is artificially disrupted using a procedure known as assisted hatching (AH), an additional technique [10]. This case study focuses on couples with a consanguineous marriage history and unsuccessful IUI and IVF-ET cycles. To overcome the prior unsuccessful IVF procedure, mechanical hatching was carried out, which produced a successful clinical pregnancy.

## Case presentation

Patient information

A 32-year-old female patient and her 37-year-old spouse had been trying to get pregnant for at least six years and visited an IVF center in Wardha. The pair had a history of consanguineous marriage. The couple began trying to conceive in 2010, and from 2013 to 2017, they underwent IUI and IVF-ET treatments. After all these years of ongoing tries, they had four unsuccessful IUI efforts and one unsuccessful IVF-ET cycle. The female partner had a regular menstrual cycle. Neither partner had ever smoked or taken drugs or alcohol. Neither of the patients had a family history of genetic abnormalities, other medical conditions, or previous therapy. The male patient did not exhibit any addiction-related habits. Normal sperm parameters were found in the male partner's semen after examination.

Clinical findings

Semen analysis had been performed on the male partner. A sample of semen was collected in the laboratory. The sperm testing of semen, according to WHO, revealed normal sperm parameters. The total sperm count was 86 million/mL, with 85% of total motility, as shown in Table 1. The female spouse underwent a blood test to measure hormone levels, which all resulted within normal limits, as shown in Table 2.

**Table 1:** Semen analysis report

Parameters	Values	Lower reference limits
Total sperm count	86 million/mL	39%
Total motility	85%	40%

**Table 2:** Blood hormone test

Hormones	Levels	Lower reference limits
Luteinising hormone (LH) mIU/mL	2.56	2-10
Follicle stimulating hormone (FSH) mIU/mL	4.96	3-9
Anti-Müllerian hormone (AMH) ng/mL	0.63	0.7-3.5

The uterine and fallopian tubes were examined using hysterosalpingography to see whether they were blocked. Everything appeared to be fine after the procedure. The follicular investigation was carried out using transvaginal ultrasound. Antral follicle count (AFC) was within normal limits. Endometrium thickness was average. No history of problems was noted with endometrium thickness in the female partner.

Timeline

The couple married in 2009 and began trying to conceive from 2010 to 2013. The female partner began her journey toward fertility treatment after experiencing difficulties conceiving naturally. In June 2013, as the first treatment option by the healthcare provider, the patient and her partner decided to pursue IUI with a fresh semen sample. The first IUI cycle was initiated in August 2013. Unfortunately, the first IUI cycle did not result in a successful pregnancy. In February 2014, a second IUI cycle was attempted. Regrettably, the second IUI cycle ended without achieving a positive pregnancy result. The patient and her partner continued to seek guidance from their healthcare providers regarding alternative fertility treatments. The third IUI cycle was undertaken in August 2014. Despite the patient's unwavering efforts, the third IUI cycle ended in disappointment. Again, in January 2015, the fourth IUI cycle was pursued. Like the previous attempts, the fourth IUI cycle did not lead to a successful pregnancy. After multiple unsuccessful IUI cycles, the decision was made to transition to a more advanced fertility treatment IVF in June 2015. In August 2015, the first IVF-ET cycle was performed. Unfortunately, the first IVF-ET cycle did not result in a positive pregnancy outcome.

After all these failed attempts, the patient and her partner took time to regroup and consult with the medical team of an IVF center in Wardha about further steps in March 2016. Initial preparations, including semen analysis, blood hormone test, ovarian stimulation, and egg retrieval, were carried out. The first IVF-ET cycle in the IVF center in Wardha was attempted at the end of May 2016. Despite the patient's perseverance and the collective efforts of the medical team, the first IVF-ET cycle ended without achieving a successful pregnancy. Additional tests and evaluations were conducted to identify potential factors contributing to implantation failure. In January 2017, a second IVI-ET cycle was planned.

Diagnostic assessment

The history of implantation failure included four unsuccessful IUI cycles and one unsuccessful IVF-ET cycle. The patient underwent an Endometrial Receptivity Analysis (ERA) test. ERA test is a diagnostic evaluation utilised in the area of ART, especially IVF-ET. The best time to transfer an embryo is during the window of implantation, identified by the ERA test, to increase the likelihood of successful implantation and pregnancy. The test recommended performing the ET 16 hours later than the time at which this endometrial biopsy was performed.

Therapeutic intervention

The patient was consulted for an IVF cycle. The male partner’s sperm was cryopreserved. The female partner underwent ovarian stimulation by the gonadotropin-releasing hormone (GnRH) antagonist method. The GnRH antagonist method advised 150 IU of recombinant follicle stimulating hormone (FSH) from day three of the menses cycle with 0.25 mg of GnRH antagonist per day until the follicles reached 17 mm in diameter with regular monitoring of follicular growth. When the bristles reached a size of ≥ 17 mm in diameter, a human chorionic gonadotropin (HCG) of 10,000 IU was administrated. After 35 hrs, the oocyte was retrieved. One germinal vesicle (GV) stage oocyte, two M1 phase oocytes, and 10 M2 phase oocytes were recovered out of 13 oocytes. Intracytoplasmic sperm injection was performed. Four day-five blastocysts were formed. All four were cryopreserved in the 2+2 group. After two months, the patient underwent a two day-five frozen ET. On the following 14th day, β HCG level was checked, which showed negative results with β HCG level of 8.7 μl/mL.

The ERA test was performed after the previous implantation failure to check the ideal time for ET. Again, after two months, the patient was consulted for mechanical hatching. This process involved creating a small hole or thinning a portion of the ZP using specialised micromanipulation tools or lasers. This weakening of the ZP can make it easier for the embryo to hatch.

**Figure 1:**
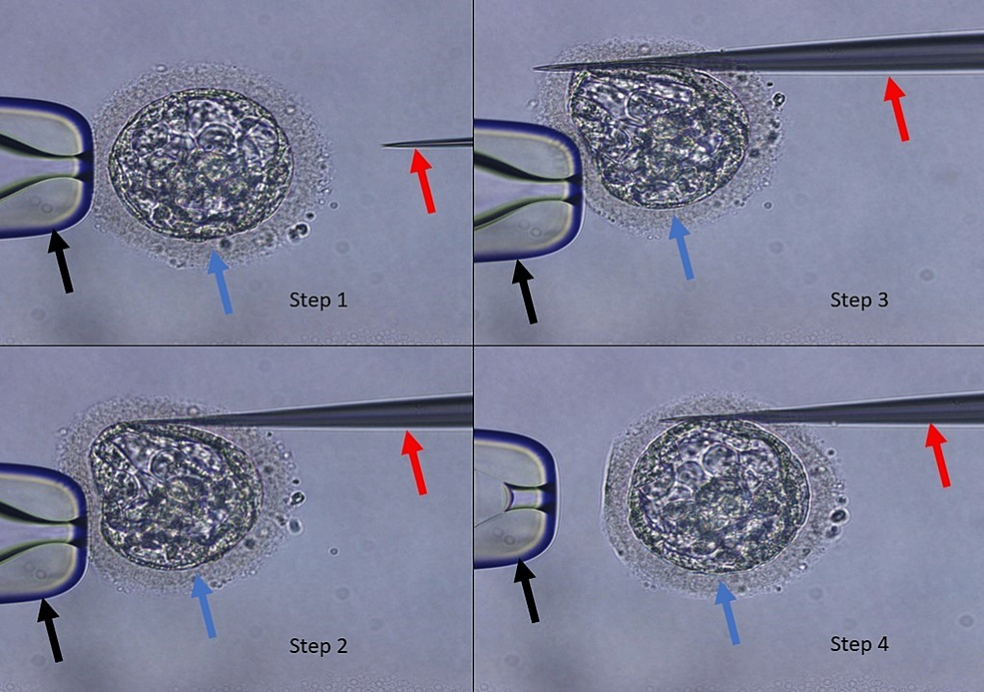
Process of mechanical hatching using PZD pipettes PZD: partial zona dissection

This mechanical hatching was performed with one frozen-thawed day-five embryo (blue arrow) using a partial zona dissection (PZD) pipette (red arrow). The embryo was stabilised using a holding pipette (black arrow). A PZD pipette was pushed tangentially into the area between the blastomeres and the ZP until it again pierced through the ZP. The embryo was released from the holding pipette (Figure 1). The other frozen-thawed day-five embryo was kept in hyaluronic acid and incubated for 10 minutes before ET. Both of the embryos were transferred to the uterine cavity of the patient. This time, one embryo got implanted, and β HCG. The level was 202 μl/mL on the 12th day of ET, which revealed successful implantation. Controlled ovarian stimulation, egg harvesting, and embryo culture were all performed by conventional guidelines during the IVF process. Encouraging results were seen during clinical monitoring after ET.

Follow-up and outcomes

After ET, the patient was advised to avoid strenuous activities and rest properly. Progesterone, iron, and other supplements were recommended for her, and a pregnancy test check was requested after 14 days. Urine pregnancy test (UPT) confirmed positive on the 12th day after ET; after this, the β HCG level was 202 μl/mL. After three weeks, ultrasound tests were planned to track the pregnancy progression and ensured that the embryo had been implanted correctly in the uterus.

## Discussion

The positive outcome of this case study emphasises the possible effectiveness of mechanical hatching as a valuable strategy for resolving issues related to repeated implantation failure. This shows how crucial it is to choose the right technologies based on each patient's unique needs. Furthermore, the fact that mechanical hatching worked in this instance raises questions about its potential for use in ART. Couples struggling with infertility might find hope in knowing the circumstances that lead to implantation failure and adjusting therapies accordingly, which can significantly increase the likelihood of a healthy pregnancy.

According to Fehilly et al., AH has been an option for elderly patients, those who have frozen-thawed embryos, and those who have had repeated IVF-ET failure [11]. In the 1980s, AH, which intentionally disturbs the ZP, was first proposed. On a similar line, Antinori et al. conducted a study that stated numerous strategies have been suggested and implemented to increase the implantation rate [12]. These included enhancing the ET procedure, endometrial receptivity, and the embryo's ability to implant. In the study by Hammadeh et al., there was evidence that AH had a benefit in some circumstances, such as for older women over the age of 38 and inpatient groups with poor outcomes (such as those who have had two or more failed IVF cycles and low embryo quality) [13]. An increased rate of clinical pregnancy and implantation followed AH. This research formed the basis of our application of mechanical hatching in our case. The limitation of this case report is that it is performed on a single patient, and the result of this report cannot be generalised to the population. Also, it is essential to note that ARTs are continually evolving, and techniques like mechanical hatching are subject to ongoing research and refinement [14].

Increasing female age has been linked to chromosomal aneuploidy in embryos, which can cause implantation issues, miscarriages, and the birth of a child with a condition [15]. For couples in consanguineous marriages with fertility issues, the close genetic relationship between the partners might contribute to a higher likelihood of passing on certain genetic factors that could interfere with successful conception and pregnancy [15,16]. This case report featured a patient who consistently had implantation failure. The woman was referred for her eight-year primary infertility, which included four unsuccessful IUIs and one unsuccessful IVF round. The patient was consulted for ICSI with mechanical hatching of the day five embryo, resulting in a positive pregnancy.

Numerous embryos possessed variability in zona thickness early in their development, demonstrating that zona thinning is a dynamic process [13]. It has been shown that the ZP thickness and patients are related. The main goal of AH was to increase the embryo’s chances of successfully implanting in the uterine lining, mainly when repeated miscarriages or abortions have been a problem [17].

It is important to remember that AH is only selectively advised for some individuals undergoing IVF. The decision to use AH should be made after a thorough review by the fertility specialist, considering factors including the woman’s age, the outcomes of earlier IVF cycles, and the overall quality of the embryo. The medical staff must carefully assess the procedure’s usage since there are additional possible hazards, such as harm to the source.

## Conclusions

This case report details the successful use of mechanical hatching on the embryo before ET in the case of a patient who had been dealing with primary infertility for eight years and had a history of consanguineous marriage and recurrent implantation failure. The case above further emphasizes how mechanical hatching may benefit individuals who have experienced repeated implantation failure. The findings suggest that tailored interventions such as mechanical hatching may hold promise for addressing specific challenges in ART, emphasizing the importance of individualized approaches in the evolving landscape of reproductive technologies.
